# Kombucha SCOBY as a Fermentation-Derived Biofilm Matrix: Species-Resolved Microbial Communities and Multidimensional In Vitro Bioactivities

**DOI:** 10.3390/polym18060764

**Published:** 2026-03-20

**Authors:** Anita Hartono, Kyra Singgih Palupi, Riza-Arief Putranto, Antonello Santini, Fahrul Nurkolis

**Affiliations:** 1 Mambucha Scobylogy International, Jakarta 14240, Indonesia; 2 Biology Department, Faculty of Science and Technology, State Islamic University of Sunan Kalijaga (UIN Sunan Kalijaga), Yogyakarta 55281, Indonesia; 3 Department of Biotechnology, Faculty of Medical Sciences, Esa Unggul University, Jakarta 11510, Indonesia; 4 Indonesian Oil Palm Research Institute, Medan 20158, Indonesia; 5 Department of Pharmacy, University of Napoli Federico II, Via Domenico Montesano, 49, 80131 Napoli, Italy; 6 Institute for Research and Community Service, State Islamic University of Sunan Kalijaga (UIN Sunan Kalijaga), Yogyakarta 55281, Indonesia; fahrul.nurkolis.mail@gmail.com; 7 Master of Basic Medical Science, Faculty of Medicine, Universitas Airlangga, Surabaya 60131, Indonesia; 8 Medical Research Center of Indonesia, Surabaya 60281, Indonesia

**Keywords:** kombucha SCOBY, full-length 16S–ITS sequencing, Oxford Nanopore Technology, antioxidant activity, α-glucosidase and DPP4 inhibition, anti-aging enzymes

## Abstract

Kombucha fermentation is driven by a Symbiotic Culture of Bacteria and Yeast (SCOBY), a cellulose-rich biofilm that hosts a complex microbial consortium. While most kombucha studies focus on the liquid beverage, the SCOBY pellicle itself remains underexplored, particularly with respect to species-level microbial resolution and its intrinsic biological activities. In this study, a commercial kombucha SCOBY was characterized using full-length 16S rRNA gene and ITS amplicon sequencing based on Oxford Nanopore Technology, enabling species-level taxonomic resolution. In parallel, hydroalcoholic and aqueous extracts of dried SCOBY biomass were evaluated for in vitro antioxidant activity (DPPH and ABTS assays), antidiabetic-related enzyme inhibition (α-glucosidase and dipeptidyl peptidase-4, DPP4), and anti-aging-related enzyme inhibition (tyrosinase and elastase). The SCOBY bacterial community was strongly dominated by acetic acid bacteria, with *Komagataeibacter saccharivorans* and *Acetobacter tropicalis* accounting for more than 60% of total reads, reflecting a biofilm structure optimized for cellulose production and oxidative metabolism. The yeast community showed marked unevenness, with *Brettanomyces bruxellensis* representing over 80% of reads, consistent with its known role in ethanol production and stress tolerance within kombucha systems. In vitro assays revealed that hydroalcoholic SCOBY extracts consistently exhibited higher biological activity than aqueous extracts across all tested assays. However, both extracts showed substantially lower potency than purified reference compounds, indicating moderate but measurable bioactivity typical of complex fermented matrices. These findings support the potential valorization of SCOBY as a fermentation-derived biomaterial and functional ingredient while underscoring the need for further chemical characterization, mechanistic studies, and biological validation beyond enzyme-based assays.

## 1. Introduction

Kombucha is a traditional fermented beverage produced through the metabolic interplay of a symbiotic culture of bacteria and yeast (SCOBY), a cellulose-rich biofilm that develops at the air–liquid interface during fermentation [1]. The SCOBY is not merely a microbial carrier but represents a highly organized fermentation matrix in which microorganisms are spatially embedded within bacterial cellulose and continuously interact through metabolic cross-feeding [2]. Acetic acid bacteria (AAB), particularly members of the genus *Komagataeibacter*, are major contributors to cellulose biosynthesis and organic acid production, while yeasts ferment sugars into ethanol and other intermediates that sustain bacterial metabolism [3,4]. This mutualistic relationship governs the structural integrity, biochemical composition, and functional properties of kombucha fermentation [5,6].

Despite the rapid growth of kombucha research in recent years, most microbiological investigations have centered on the liquid phase of the beverage, primarily examining fermentation-driven changes in microbial populations, organic acid profiles, and sensory characteristics [7,8,9,10,11]. The SCOBY pellicle constitutes the central functional unit of kombucha fermentation, as it houses the biofilm-associated microbial consortium that drives continuous cellulose production, oxidative metabolism, and long-term microbial stability [8,11,12,13]. Despite the co-occurrence of the SCOBY and the fermentation liquid, their microbial communities may diverge substantially; however, systematic characterization of SCOBY-associated microbiota remains limited [14,15].

The widespread use of short-read approaches that sequence only partial regions of the 16S rRNA gene and fungal ITS often restricts identification to the genus level, a limitation of particular relevance to AAB because closely related *Komagataeibacter* species may share near-identical 16S regions despite marked divergence in cellulose production, acidification kinetics, and stress tolerance [16,17,18,19,20]. Frequently reported kombucha yeasts, including *Brettanomyces*, *Zygosaccharomyces*, and *Pichia*, exhibit species-level heterogeneity in fermentation performance and metabolic output that is not robustly captured by short-amplicon approaches alone [21,22,23,24,25,26].

Recent advances in long-read sequencing technologies, especially Oxford Nanopore Technologies (ONT), enable full-length 16S rRNA gene and complete ITS region sequencing, providing accurate species-level identification in complex microbial ecosystems [27]. Leveraging full-length 16S–ITS sequencing for kombucha SCOBY can refine characterization of the biofilm matrix [13,28] by resolving community composition across abundance tiers and linking key taxa to potential functional roles during fermentation [12]. This species-level clarity is increasingly important for establishing reproducible starter cultures and implementing robust quality assurance frameworks for kombucha manufacturing [29].

On the other hand, kombucha has been widely studied for its microbial consortia and, increasingly, for reported biological activities such as antioxidant, antidiabetic, and anti-aging potentials [30,31,32]. These functional attributes are frequently ascribed to fermentation-associated chemistry, including accumulation of organic acids, biotransformation of polyphenols, and production of microbial secondary metabolites [1,15,33,34,35]. The evidence for the SCOBY pellicle remains comparatively understudied, as a fermentation-derived biomaterial is well established [32,36]. The SCOBY matrix contains a bacterial cellulose network that entraps microbial cells and retains fermentation products, suggesting that the pellicle itself could exhibit measurable in vitro bioactivities [32,36,37].

In vitro antioxidant assays, including 2,2-diphenyl-1-picrylhydrazyl (DPPH) and 2,2′-azino-bis(3-ethylbenzothiazoline-6-sulfonic acid) (ABTS) radical scavenging tests, are widely employed to assess the capacity of fermented matrices to neutralize free radicals [31,32]. For antidiabetic-related mechanisms, inhibition of α-glucosidase provides insight into reduced carbohydrate hydrolysis, while inhibition of dipeptidyl peptidase-4 (DPP4) is relevant to incretin turnover and postprandial glucose regulation [30,31]. Furthermore, inhibition of tyrosinase and elastase is frequently applied as an in vitro proxy for anti-aging or cosmetic-relevant potential [30,37]. Evaluating these complementary in vitro assays allows for a multidimensional assessment of SCOBY bioactivity without implying direct clinical efficacy.

Importantly, the relationship between SCOBY microbial composition and its in vitro biological activities remains poorly understood. Integrating high-resolution microbial profiling with functional bioactivity assessment may help elucidate how dominant microbial species and fermentation-derived structures contribute to antioxidant capacity, enzyme inhibition, and overall functional potential [24]. Such an integrated approach aligns with current interests in fermentation science, where microbial ecology, process control, and functional outcomes are increasingly studied together [38].

Therefore, the objectives of this study were to characterize the bacterial and yeast communities present in a kombucha SCOBY pellicle at species-level resolution using full-length 16S rRNA and ITS amplicon sequencing based on ONT and to evaluate the in vitro biological activities of SCOBY extracts, including antioxidant activity (DPPH and ABTS), antidiabetic-related enzyme inhibition (α-glucosidase and DPP4), and anti-aging enzyme inhibition (tyrosinase and elastase). By focusing on the SCOBY biomass rather than the liquid kombucha, this study provides a comprehensive view of the microbial architecture and functional potential of the fermentation matrix itself, with implications for SCOBY valorization, fermentation control, and the development of standardized kombucha starter systems.

## 2. Materials and Methods

### 2.1. SCOBY Source and Sample Description

Kombucha SCOBY pellicles were obtained from Mambucha Scobylogy International (Jakarta, Indonesia). The SCOBY was produced from a commercial kombucha formulation containing green tea, black tea, jasmine flower, cane sugar, and water; the complete formulation was not disclosed due to intellectual property protection (patent is under application), as the product is commercially available. SCOBY pellicles were harvested at day 14 of fermentation (pH = 3.2) and transported to the laboratory under chilled conditions for immediate processing.

### 2.2. SCOBY Preparation and Low-Temperature Oven Drying

Upon arrival, SCOBY pellicles were separated from the fermentation liquor and gently rinsed 2–3 times with chilled distilled water to remove residual tea components and soluble sugars adhering to the surface. The pellicles were blotted with sterile paper towels to remove excess water and cut into small pieces (approximately 0.5–1.0 cm). The material was then dried in a forced-air oven at 40–45 °C until a constant weight was achieved. Low-temperature oven drying was selected to achieve controlled dehydration while minimizing degradation of heat-sensitive metabolites, as bacterial cellulose remains structurally stable at this temperature range, and gradual moisture removal helps preserve matrix integrity. Dried SCOBY was ground into a fine powder using a clean Mortar Grinder MG200 (Beijing Grinder Instrument Co., Ltd., Beijing, China) and passed through a standard sieve (40 mesh; ASTM E11/ISO 3310-1 (https://store.astm.org/standards/e11, https://www.iso.org/standard/62410.html, accessed on 10 October 2025)). The powder was stored in airtight, light-protected containers at −20 °C until extraction.

### 2.3. Preparation of Hydroalcoholic and Aqueous Extracts

Two extracts were prepared from the dried SCOBY powder: a hydroalcoholic extract and an aqueous extract.

#### 2.3.1. Hydroalcoholic Extraction

Dried SCOBY powder was mixed with 70% (*v*/*v*) ethanol (Sigma-Aldrich^®^, MilliporeSigma/Merck Life Science; Burlington, MA, USA/Darmstadt, Germany) at a solid-to-solvent ratio of 1:20 (*w*/*v*) (10 g powder in 200 mL solvent). The mixture was extracted under agitation on an orbital shaker (150 rpm) at room temperature for 16–24 h in the dark. Extracts were centrifuged (10,000× *g*, 10 min), and the supernatant was collected. The residue was re-extracted once under identical conditions, and supernatants were pooled. The combined filtrate was concentrated under reduced pressure (rotary evaporation) at ≤40 °C, then dried to constant mass (or further dried under a gentle nitrogen stream) to yield the crude hydroalcoholic extract. The dried extract was stored at −20 °C in amber vials.

#### 2.3.2. Aqueous Extraction

Dried SCOBY powder was extracted with distilled water at 1:20 (*w*/*v*). The mixture was heated at 80 °C for 2 h with intermittent mixing, cooled to room temperature, and centrifuged (10,000× *g*, 10 min). The elevated extraction temperature was selected to enhance the release of water-soluble and matrix-bound compounds from the dense cellulose network, as increased temperature improves mass transfer and extraction efficiency in polysaccharide-rich biomaterials. This approach also reflects conventional hot-water extraction practices used for plant and fermentation-derived materials. However, thermal treatment at 80 °C may induce partial degradation or structural transformation of certain heat-sensitive metabolites, which could influence the measured biological activities. The supernatant was collected, and the residue was re-extracted once. Pooled aqueous extracts were filtered and then freeze-dried (VaCo Series freeze dryer, Zirbus Technology GmbH, Bad Grund/Harz, Germany) or oven-dried at 45 °C to obtain the crude aqueous extract powder. Extracts were stored at −20 °C until analysis.

#### 2.3.3. Preparation of Stock Solutions and Working Dilutions

For DPPH and ABTS (Sigma-Aldrich^®^, MilliporeSigma/Merck Life Science; Burlington, MA, USA/Darmstadt, Germany), extracts were dissolved in methanol (MeOH; Sigma-Aldrich^®^, MilliporeSigma/Merck Life Science; Burlington, MA, USA/Darmstadt, Germany) to prepare stock solutions (10 mg/mL) and serially diluted to the desired concentrations. For enzyme-based assays (α-glucosidase, DPP4, tyrosinase, elastase), extracts were dissolved in dimethyl sulfoxide (DMSO) to prepare concentrated stocks and then diluted with the respective assay buffers such that the final DMSO concentration in wells was kept low (commonly ≤1% *v*/*v*) to minimize enzyme inhibition by solvent. All measurements were performed at least in triplicate. Recombinant α-glucosidase, tyrosinase, and elastase enzymes were purchased from Sigma-Aldrich (Merck MilliporeSigma; Burlington, MA, USA) and used without further purification for in vitro enzymatic assays. Solvent-only controls (enzyme + buffer + DMSO at equivalent final concentration without extract) were included in all enzyme assays to verify that the solvent (≤1% *v*/*v*) did not contribute to measurable enzyme inhibition.

### 2.4. Antioxidant Assays

#### 2.4.1. DPPH Radical Scavenging Assay

DPPH radical scavenging activity was measured using a standard spectrophotometric method with minor modifications [39]. Briefly, DPPH solution (0.1 mM) was prepared in methanol and protected from light. In a 96-well plate, extract solutions at various concentrations were mixed with DPPH solution and incubated for 30 min at room temperature in the dark. Absorbance was read at 517 nm using a microplate reader (BioTek Synergy HTX Multi-Mode Reader, Agilent BioTek Instruments, Winooski, USA). Trolox and L-ascorbic acid (vitamin C; Sigma-Aldrich, Merck KGaA, Burlington, MA, USA/Darmstadt, Germany) were used as positive controls. Methanol served as the blank, while the DPPH solution without a sample served as the negative control. Radical scavenging activity was calculated as

$$\%\;\mathrm{Inhibition}=\left(1-\frac{A_{\mathrm{sample}}}{A_{\mathrm{control}}}\right)\times 100$$

where $A_{\mathrm{control}}$ is the absorbance of DPPH without the sample, and $A_{\mathrm{sample}}$ is the absorbance in the presence of the extract. The IC_50_ values were determined by nonlinear regression from concentration–response curves.

#### 2.4.2. ABTS Radical Cation Scavenging Assay

The ABTS radical cation (ABTS^+^) was generated by mixing ABTS (7 mM) with potassium persulfate (2.45 mM) and incubating the mixture for 12–16 h in the dark at room temperature. The ABTS^+^ working solution was diluted with phosphate-buffered saline (PBS) to obtain an absorbance of 0.70 ± 0.02 at 734 nm. In a 96-well plate, diluted ABTS^+^ solution was mixed with sample solutions and incubated for 10 min at room temperature. Absorbance was measured at 734 nm using a microplate reader (BioTek Synergy HTX, Agilent BioTek Instruments, Winooski, VT, USA). Trolox and vitamin C were used as positive controls. Percent inhibition and IC_50_ values were calculated as described above [40].

### 2.5. Antidiabetic Enzyme Inhibition Assays

#### 2.5.1. α-Glucosidase Inhibition Assay

α-glucosidase inhibitory activity was evaluated using a chromogenic substrate assay [41,42]. Briefly, α-glucosidase enzyme solution was prepared in phosphate buffer (pH neutral), and p-nitrophenyl-α-D-glucopyranoside (pNPG; Sigma-Aldrich, Merck KGaA) was used as the substrate. In a 96-well plate, extracts at various concentrations were pre-incubated with α-glucosidase for 10 min at 37 °C, followed by the addition of pNPG to initiate the reaction. After incubation (30 min), the reaction was stopped by adding sodium carbonate solution, and absorbance was measured at 405 nm. Acarbose (Sigma-Aldrich; Merck KGaA, Darmstadt, Germany) served as the positive control. Enzyme activity (%) and inhibition (%) were calculated relative to a control reaction containing enzyme and substrate without extract. Appropriate sample blanks (extract + substrate without enzyme) were included to correct for extract color/turbidity. IC_50_ values were obtained from concentration–response curves.

#### 2.5.2. DPP4 Inhibition Assay

DPP4 inhibitory activity was assessed using a commercially available DPP4 activity assay kit or a standard fluorometric/chromogenic substrate method, following the manufacturer’s instructions (Merck MilliporeSigma; Burlington, MA, USA) [43]. Briefly, the DPP4 enzyme was incubated with sample solutions for 15 min at 37 °C, and the reaction was initiated by adding the DPP4 substrate. After incubation, fluorescence was measured at an excitation wavelength of 360 nm and an emission wavelength of 460 nm, according to the manufacturer’s instructions. Sitagliptin (Sigma-Aldrich, Merck KGaA) was used as the positive control. Negative controls (enzyme + substrate without inhibitor) and sample blanks (extract + substrate without enzyme) were included. Percent inhibition and IC_50_ values were calculated from concentration–response curves.

### 2.6. Anti-Aging Enzyme Inhibition Assays

#### 2.6.1. Tyrosinase Inhibition Assay

Tyrosinase inhibitory activity was measured using a spectrophotometric method based on the oxidation of L-DOPA by mushroom tyrosinase [44]. Briefly, in a 96-well plate, extracts at various concentrations were pre-incubated with tyrosinase in phosphate buffer for 10 min at 25–37 °C. The substrate (L-DOPA) was added to start the reaction, and the formation of dopachrome was monitored by measuring absorbance at 475 nm. Kojic acid (Sigma-Aldrich, Merck KGaA) was used as the positive control. Sample blanks were included to correct for extract color. Percent inhibition and IC_50_ values were calculated relative to enzyme controls.

#### 2.6.2. Elastase Inhibition Assay

Elastase inhibitory activity was determined using porcine pancreatic elastase and a chromogenic substrate such as N-succinyl-Ala-Ala-Ala-p-nitroanilide (SANA; Sigma-Aldrich, Merck KGaA) or a comparable elastase substrate [45,46]. In a 96-well plate, elastase was pre-incubated with extracts for 10 min at 37 °C, then the substrate was added to initiate the reaction. The release of p-nitroaniline was monitored by absorbance at 405 nm over a fixed time. Ursolic acid (Sigma-Aldrich, Merck KGaA) served as the positive control. Controls and sample blanks were included, and percent inhibition and IC_50_ values were calculated as described above.

### 2.7. Bacterial and Yeast Communities Analysis

#### 2.7.1. Sample Collection

SCOBY samples were obtained from a commercial kombucha culture in Yogyakarta, Indonesia (Mambucha kombucha). The sample consisted of a piece of the cellulose-rich SCOBY pellicle (approximately 5 cm in diameter) taken from an active kombucha fermentation. Importantly, only the solid SCOBY mat was sampled, not the liquid tea, in order to profile the biofilm-associated community. The SCOBY biomass was rinsed briefly with sterile saline to remove residual tea liquid and then stored at 4 °C until DNA extraction.

#### 2.7.2. DNA Extraction

Genomic DNA was extracted from ~0.25 g of SCOBY wet biomass using the NucleoSpin^®^ Tissue Kit (Macherey-Nagel GmbH & Co. KG, Düren, Germany) following the manufacturer’s protocol, with modifications appropriate for mixed Gram-positive, Gram-negative, and yeast-containing samples. To ensure effective lysis of cellulose-embedded bacteria and yeasts, samples were first incubated with lysozyme (20 mg/mL final concentration; approximately 40 U per reaction) at 37 °C for 30 min, followed by lyticase treatment (200 U per reaction) at 30 °C for 45 min to facilitate yeast cell wall disruption. After enzymatic digestion, mechanical disruption was performed using bead-beating (0.1 mm zirconia/silica beads, 2 × 60 s cycles) to enhance DNA recovery from the cellulose matrix. DNA quality and quantity were checked by spectrophotometry and agarose gel electrophoresis [12,25].

#### 2.7.3. Amplicon PCR and Sequencing

Full-length 16S rRNA gene and full-length ITS region amplicons were generated using Oxford Nanopore Technologies (ONT) protocols [47]. For bacteria, the nearly complete 16S rRNA gene (~1500 bp, covering V1–V9 regions) was amplified using universal primers 27F/1492R. For fungi/yeasts, the entire internal transcribed spacer (ITS) region (including ITS1, 5.8S rRNA, and ITS2) was amplified with universal fungal primers (e.g., ITS1/ITS4). High-fidelity DNA polymerase was used to minimize PCR error. PCR products were verified on agarose gels and then purified. Amplicon libraries were prepared with ONT library prep kits and barcoding adapters according to ONT’s 16S/ITS sequencing protocol. Sequencing was performed on an ONT MinION device R10 flow cell (Oxford Nanopore Technologies Ltd., Oxford, UK), producing real-time long-read data. A single ONT flow cell was used for the 16S library and another for the ITS library.

#### 2.7.4. Bioinformatic Analysis

Basecalling of Nanopore reads was done using Guppy ONT (Oxford Nanopore Technologies, Oxford, UK; https://nanoporetech.com/software/other/guppy, accessed on 13 October 2025). Demultiplexing and adapter trimming were performed, though in this case, only one sample per run was present. For the 16S reads, we employed a pipeline that uses accurate full-length 16S classification: reads were processed with the ONT EPI2ME workflow or an equivalent custom pipeline. Briefly, chimeric reads were filtered (using Uchime or similar), and high-quality full-length 16S reads were taxonomically classified using a 16S reference database (e.g., SILVA or NCBI 16S) at 99% or 100% identity thresholds to resolve species. For the ITS reads, a similar approach was used: ITS reads were clustered or denoised and matched against a curated fungal ITS database (e.g., UNITE) for species identification. Alpha diversity indices (richness and diversity metrics) were calculated using Qiime2 (https://qiime2.org, accessed on 13 October 2025) and are reported to characterize community diversity. The relative abundance of each taxon was determined as the proportion of total classified reads in that domain (bacterial 16S or fungal ITS). Data visualization was done through Krona charts and Sankey plots provided in the sequencing report, as well as custom bar graphs.

### 2.8. Data Analysis and Reporting

All in vitro experiments were conducted at least in triplicate, and data were expressed as mean ± standard deviation (SD). Concentration–response curves were generated, and IC_50_ values were estimated by nonlinear regression. Where appropriate, results may be reported as IC_50_ (µg/mL or mg/mL) and/or inhibition (%) at selected concentrations. Statistical comparisons were performed on inhibition percentages at fixed concentrations rather than directly on IC_50_ values.

## 3. Results and Discussions

### 3.1. Species-Level Resolution of Bacterial and Yeast Communities in Kombucha SCOBY

#### 3.1.1. Bacterial Communities in Kombucha SCOBY

The bacterial profile was strongly skewed toward AAB, with *Komagataeibacter saccharivorans* comprising 46.28% of reads and *Acetobacter tropicalis* contributing 15.40%; together, these two taxa accounted for 61.68% of the community (Table 1). The remaining top taxa were also largely AAB-related (e.g., *Novacetimonas hansenii* 5.17%; *K. rhaeticus* 4.87%; *Gluconacetobacter entanii* 3.99%), and the top 10 taxa collectively represented 85.82% of reads, indicating that most bacterial signal was carried by a small set of dominant species. This dominance structure is consistent with the established SCOBY ecology in which AAB, particularly *Komagataeibacter*, typically form the core biofilm community that supports oxidative metabolism and cellulose-rich pellicle formation [13,24,29,35,48].

**Figure 1:**
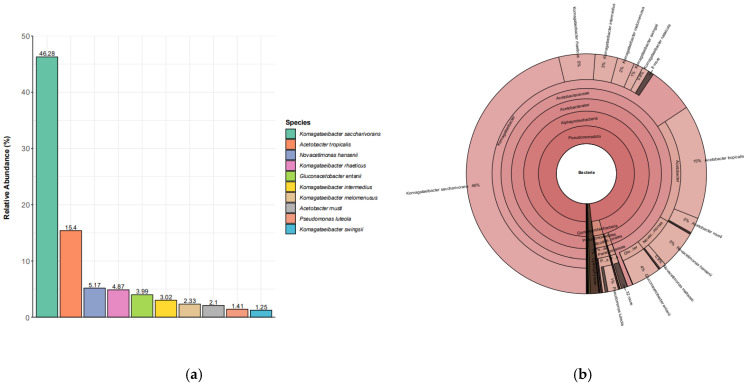

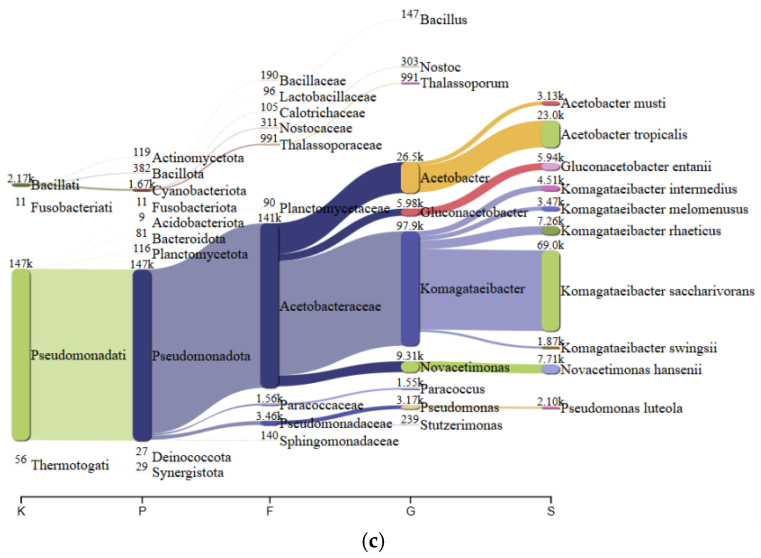
(**a**) Ten microbial species with the highest proportion of relative abundance toward total reads. The following plot presents the top 10 relative abundances at the species level. (**b**) The Krona plot revealed dominance of *Acetobacteraceae* (*Komagataeibacter saccharivorans* 60%) with *Acetobacter tropicalis* at 15% and the rest as low-abundance taxa. (**c**) The Sankey diagram traces taxonomic flow from higher to lower ranks with band width proportional to abundance, showing most reads/classifications funneling from Pseudomonadati/Pseudomonadota into *Acetobacteraceae*, dominated by *Komagataeibacter* (especially *K. saccharivorans*) with smaller contributions from *Acetobacter* (e.g., *A. tropicalis*) and other low-abundance taxa.

The high relative abundance of *Komagataeibacter* spp. aligns with their documented roles in bacterial cellulose biosynthesis and organic acid production, both of which are central to SCOBY architecture and fermentation stability (Figure 1) [13,49,50]. Across kombucha systems, AAB dominance is commonly reported in pellicle-associated communities and is often linked to oxygen-exposed biofilm growth at the air-to-liquid interface, where AAB can efficiently oxidize ethanol and sugars produced upstream by yeasts [12,13]. Species-level resolution matters here because closely related AAB can differ in cellulose yield and stress tolerance, which may translate into differences in pellicle thickness, acidification kinetics, and robustness under variable household or industrial conditions [17,49].

**Figure 2:**
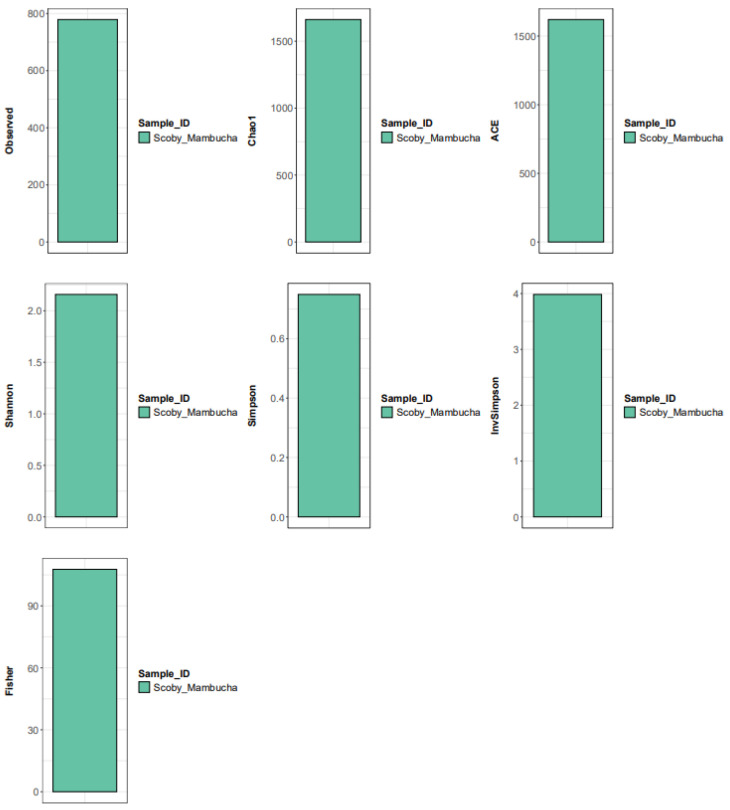
The SCOBY Mambucha sample shows 779 observed taxa of bacteria, with richness estimators (Chao1 1661.75; ACE 1620.22; Fisher 107.70) suggesting substantial unseen diversity, while diversity/evenness metrics (Shannon 2.16; Simpson 0.75; InvSimpson 3.98) indicate a moderately diverse community with noticeable dominance by a few taxa.

Alpha-diversity metrics (observed 779, Chao1 1661.75, ACE 1620.22, Shannon 2.16, Simpson 0.75, InvSimpson 3.98, Fisher 107.70) indicate a community with substantial taxonomic richness and moderate diversity, despite the dominance of a few highly abundant taxa. The large gap between observed richness and the Chao1/ACE estimators suggests the presence of considerable low-abundance diversity, consistent with a long-tail distribution underlying the dominant AAB structure (Figure 2) [7,25]. Although Shannon and Simpson values indicate measurable diversity, the strong relative abundance skew toward *Komagataeibacter* spp. remains ecologically prominent, supporting a structured community in which dominant taxa likely drive primary metabolic functions while numerous low-abundance taxa may represent niche specialists or transient members. The detection of *Pseudomonas luteola* (1.41%) warrants cautious interpretation, as low-abundance *Proteobacteria* in single-sample microbiome analyses may reflect either minor community members or background contamination; without technical controls or biological replicates, stable colonization cannot be definitively distinguished from incidental DNA signals.

#### 3.1.2. Yeast Communities in Kombucha SCOBY

The yeast community showed extreme dominance by a single species, with *Brettanomyces bruxellensis* accounting for 81.5% of reads and *Meyerozyma guilliermondii* contributing 13.31%; together they comprised 94.81%, and the top 10 taxa summed to 98.98% (Table 2). This high concentration of read support in one taxon indicates a strongly uneven structure, which is frequently observed in kombucha ecosystems where one or a few yeast species dominate sucrose hydrolysis and ethanol production, while others remain at low abundance [29,35,51]. Notably, *Brettanomyces bruxellensis* has been repeatedly reported as a major kombucha yeast across culture-based and sequencing studies [24,26,29,52], supporting the plausibility of its dominance in a SCOBY-associated biofilm context [25].

**Figure 3:**
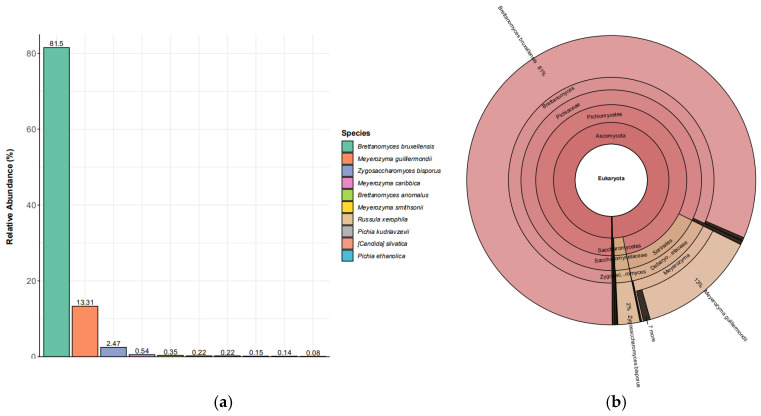

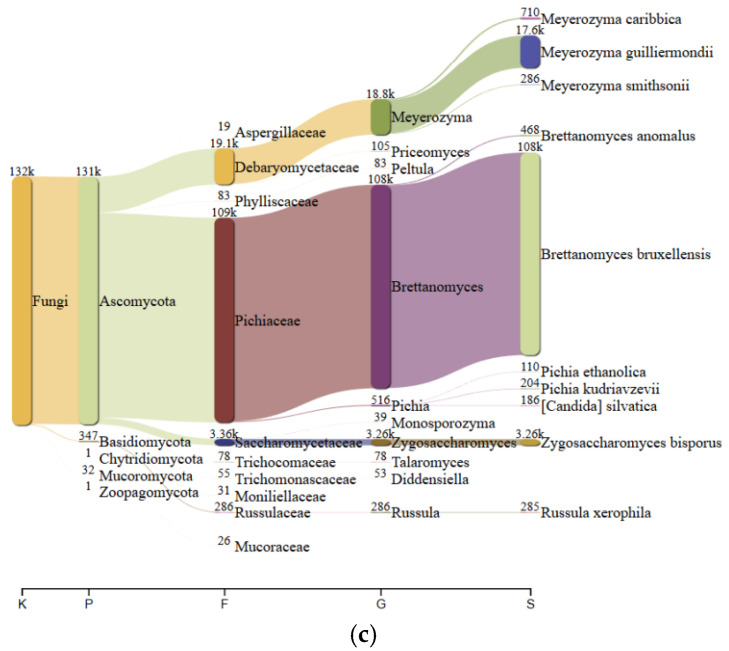
(**a**) Ten yeast species with the highest proportion of relative abundance toward total reads. The following plot presents the top 10 relative abundances at the species level. (**b**) The Krona plot indicated that the community is largely driven by *Brettanomyces bruxellensis* with *M. guilliermondii* as the main secondary species. (**c**) The Sankey diagram summarizes the taxonomic flow from higher to lower ranks with band width proportional to read support, showing most reads funneling through Fungi/Ascomycota into *Pichiaceae*, dominated by *Brettanomyces* → B. *bruxellensis*, with smaller branches toward *Meyerozyma* and other low-abundance genera/species. The legend K to S represents kingdom, phylum, family, genus, and species.

From a functional standpoint, *B. bruxellensis* dominance is biologically consistent with SCOBY cross-feeding (Figure 3a): yeasts generate ethanol and other intermediates that AAB subsequently oxidize into organic acids, reinforcing the acidic, oxygen-exposed environment that favors AAB persistence and biofilm development [13,35,51]. *Brettanomyces* spp. are also known for stress tolerance (including low pH) and for contributing to fermentation aroma through the formation of volatile compounds, traits that can support persistence within the SCOBY matrix across back-slopping cycles [53,54,55,56,57]. Although DNA-based relative abundance cannot confirm metabolic activity, a community dominated by *Brettanomyces* is directionally consistent with a fermentation network in which ethanol production and downstream oxidative metabolism are tightly coupled [13,35,58,59,60].

The prominence of *M. guilliermondii* (13.31%) is less consistently reported as a canonical kombucha yeast compared with *Brettanomyces* or *Zygosaccharomyces* [9], and it may reflect source-specific inputs (e.g., raw materials, processing environment, or local microbiota) or selective advantages under the studied conditions [35]. *Zygosaccharomyces bisporus* (2.47%), while much lower, fits with prior kombucha literature noting *Zygosaccharomyces* spp. as recurrent members of the consortium, often associated with sugar-rich, acidic fermentations. Overall, the yeast profile indicates a SCOBY in which fermentative capacity is likely concentrated in a narrow set of taxa, which may contribute to batch-to-batch stability in core outputs (e.g., ethanol precursor supply) but could also reduce ecological buffering if dominant taxa fluctuate [12].

**Figure 4:**
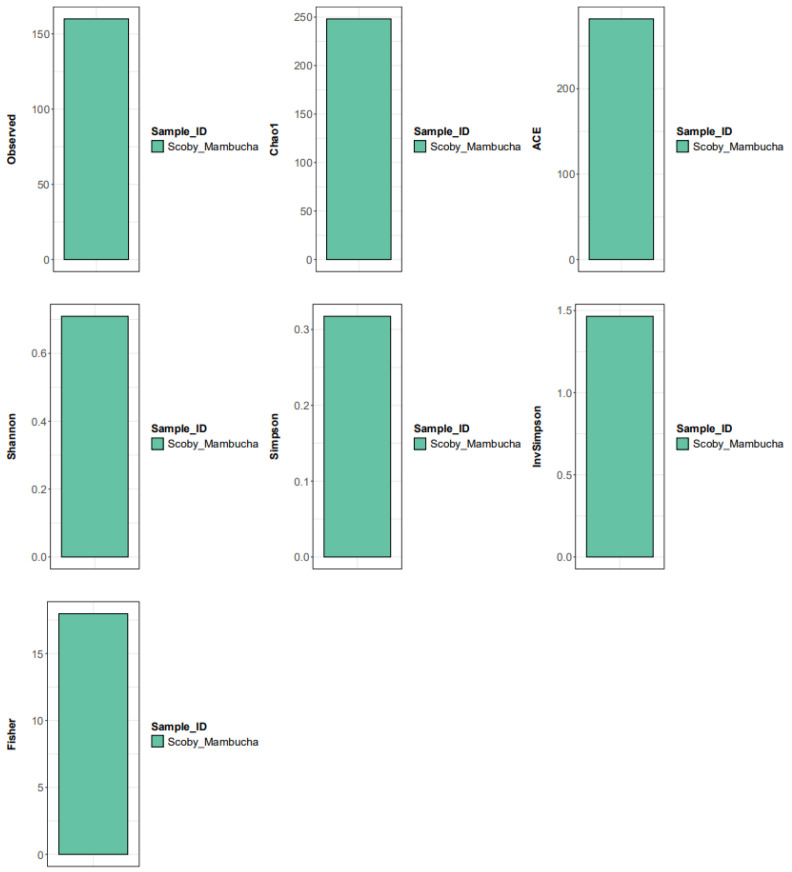
The SCOBY Mambucha sample shows 160 observed taxa of yeast, with richness estimators (Chao1 248.14; ACE 281.84; Fisher 17.98) suggesting substantial unseen diversity, while diversity/evenness metrics (Shannon 0.71; Simpson 0.32; InvSimpson 1.47) indicate a moderately diverse community with noticeable dominance by a few taxa.

Alpha-diversity metrics (observed 160, Chao1 248.14, ACE 281.84, Shannon 0.71, Simpson 0.32, InvSimpson 1.47, Fisher 17.98) support a community with low evenness (low Shannon/Simpson) and substantial unobserved richness (Chao1/ACE > observed), which is compatible with a “few-dominant + long-tail” structure (Figure 4) [7,25]. In practical terms, this pattern suggests that the SCOBY may be functionally driven primarily by dominant AAB taxa, while a broader low-abundance background could represent transient taxa, environmental carryover, or niche specialists [61]. The detection of *Pseudomonas luteola* (1.41%) also warrants cautious interpretation because low-level Proteobacteria can reflect background contamination or minor community members; without technical controls and replicate SCOBYs, it is not possible to distinguish stable colonizers from incidental DNA signals [62,63].

### 3.2. In Vitro Analysis

#### 3.2.1. Antioxidant Activity (DPPH and ABTS)

In the DPPH assay, the hydroalcoholic extract achieved 50% radical scavenging at 92.4 ± 4.8 µg/mL, whereas the aqueous extract required 168.7 ± 7.9 µg/mL (an 82.6% higher IC_50_ for the aqueous extract; Table 3). Relative to their respective reference antioxidants, the hydroalcoholic extract’s DPPH IC_50_ was 4.28× higher than Trolox (21.6 ± 1.2 µg/mL), and the aqueous extract’s DPPH IC_50_ was 8.93× higher than vitamin C (18.9 ± 0.9 µg/mL), indicating substantially lower potency than purified standards in this assay format. In the ABTS assay, IC_50_ values were 74.2 ± 3.6 µg/mL (hydroalcoholic) and 121.5 ± 6.4 µg/mL (aqueous), with the aqueous extract showing a 63.7% higher IC_50_. Compared with standards, the hydroalcoholic extract’s ABTS IC_50_ was 4.70× higher than Trolox (15.8 ± 0.7 µg/mL), while the aqueous extract’s ABTS IC_50_ was 9.07× higher than vitamin C (13.4 ± 0.6 µg/mL).

Published kombucha studies consistently emphasize that antioxidant activity is highly dependent on tea substrate, fermentation conditions, and extraction chemistry, with polyphenols and organic acids frequently implicated as major contributors [7,8,64]. Other studies profiled kombucha derived from different tea types and reported substantial variability in antioxidant-related measures across fermentation time and tea source [65,66]. In a cosmetic-oriented kombucha ferment study, DPPH IC_50_ values are reported on the order of ~10^2^ µg/mL for kombucha-containing ferments, supporting that IC_50_ magnitudes in the ~100–200 µg/mL range are plausible for complex fermented matrices rather than purified antioxidants [67]. Future work should integrate untargeted metabolomics to link microbial taxa with specific bioactive metabolites.

#### 3.2.2. Antidiabetic Enzyme Inhibition (α-Glucosidase and DPP4)

For α-glucosidase, the hydroalcoholic extract showed an IC_50_ of 112.6 ± 5.3 µg/mL, while the aqueous extract showed 198.4 ± 9.2 µg/mL (76.2% higher IC_50_ for the aqueous extract; Table 3). Against the positive control acarbose (38.9 ± 2.1 µg/mL), the hydroalcoholic extract was 2.90× higher in IC_50_, and the aqueous extract was 5.10× higher, indicating weaker inhibitory potency than the pharmaceutical comparator under these assay conditions. For DPP4, IC_50_ values were 146.8 ± 6.7 µg/mL (hydroalcoholic) and 232.1 ± 11.5 µg/mL (aqueous), a 58.1% higher IC_50_ for the aqueous extract. Compared with sitagliptin (6.2 ± 0.3 µg/mL), the hydroalcoholic extract’s IC_50_ was 23.7× higher, and the aqueous extract’s IC_50_ was 37.4× higher, consistent with markedly lower potency than a dedicated DPP4 inhibitor.

Inhibition of α-glucosidase is mechanistically aligned with reducing postprandial glucose excursions, whereas DPP4 inhibition is aligned with preserving incretin activity [68,69,70]. However, because these results are from enzyme assays using crude extracts, the IC_50_ values should be interpreted as screening-level potency rather than evidence of clinical efficacy [71,72]. A recent systematic review found very limited human evidence for kombucha health benefits, highlighting a major gap between preclinical bioactivity claims and clinical validation [73]. Recent reviews similarly summarize broad preclinical signals, including antidiabetic pathways, while also emphasizing variability in composition and the need for standardization and safety oversight [71]. Regarding α-glucosidase specifically, kombucha-related extracts have been examined as inhibitors, and solvent/extract type can materially shift apparent potency, while some studies show that tyrosinase and elastase are part of anti-aging/dermatology-relevant enzyme inhibition [74].

#### 3.2.3. Anti-Aging Activity via Tyrosinase and Elastase Inhibition

For tyrosinase, IC_50_ values were 101.3 ± 4.9 µg/mL (hydroalcoholic) and 176.9 ± 8.1 µg/mL (aqueous), with the aqueous extract requiring 74.6% higher concentration to reach 50% inhibition (Table 3). Relative to kojic acid (12.7 ± 0.6 µg/mL), the hydroalcoholic extract’s IC_50_ was 7.98× higher, and the aqueous extract’s IC_50_ was 13.9× higher, indicating weaker inhibitory activity than the reference depigmenting agent [75,76,77]. For elastase, IC_50_ values were 128.5 ± 6.1 µg/mL (hydroalcoholic) and 210.6 ± 10.3 µg/mL (aqueous), corresponding to a 63.9% higher IC_50_ for the aqueous extract. Compared with ursolic acid (9.4 ± 0.4 µg/mL), the hydroalcoholic extract’s IC_50_ was 13.7× higher, and the aqueous extract’s IC_50_ was 22.4× higher, again indicating substantially lower potency than a purified reference compound [78].

Across all in vitro assays (Table 3), the hydroalcoholic SCOBY extract showed consistently lower IC_50_ values than the aqueous extract, indicating higher apparent potency under the same assay conditions. Overall, the aqueous extract required approximately 1.58–1.83× higher concentrations than the hydroalcoholic extract to achieve 50% activity (i.e., ~58–83% higher IC_50_, depending on the assay), consistent with greater recovery of bioactive constituents into the hydroalcoholic solvent system [79,80].

### 3.3. Discussion

The present study demonstrates that the analyzed SCOBY pellicle is characterized by a strongly uneven microbial structure dominated by *Komagataeibacter saccharivorans*, *Acetobacter tropicalis*, and *Brettanomyces bruxellensis*. This “few-dominant + long-tail” configuration is consistent with ecological patterns previously described in kombucha systems, where AAB dominate the pellicle phase while yeasts provide fermentative intermediates (ethanol) that sustain oxidative metabolism in AAB [7,8,24]. The predominance of *Komagataeibacter* spp. is biologically plausible and functionally significant. Members of this genus are recognized as high-yield producers of bacterial cellulose, exhibiting robust oxidative metabolism and tolerance to acidic conditions [17,49]. Comparative genomic analyses have shown that different *Komagataeibacter* species harbor distinct cellulose synthase operons and stress-response systems, which can influence cellulose yield, crystallinity, and biofilm robustness [4,49]. Therefore, species-level resolution—enabled here by long-read sequencing—provides added value compared with short-read genus-level profiling, particularly in systems where polymer production is biologically driven.

The yeast community was overwhelmingly dominated by *Brettanomyces bruxellensis*, with *Meyerozyma guilliermondii* as a secondary contributor. This extreme skew toward a single yeast species has been reported in several kombucha ecosystems [25,26,29]. *B. bruxellensis* is known for high ethanol productivity, acid tolerance, and metabolic versatility [53,54]. Its dominance likely reinforces a stable cross-feeding network in which yeast-derived ethanol is oxidized by AAB to acetic acid and other metabolites, stabilizing the aerobic biofilm environment [13]. However, DNA-based relative abundance does not necessarily equate to metabolic activity. Previous work has shown that microbial presence and transcriptional activity may diverge substantially [58,59]. Thus, while the compositional structure supports established ecological models of SCOBY function, the current findings should be interpreted as structural rather than activity-based profiling. Integration with metatranscriptomics or metabolomics would be required to directly link dominant taxa with functional metabolite output.

The hydroalcoholic extract consistently showed lower IC_50_ values than the aqueous extract in both DPPH and ABTS assays. This difference likely reflects solvent polarity effects and the extraction efficiency of phenolic compounds, organic acids, and fermentation-derived secondary metabolites. Hydroalcoholic systems are known to enhance the recovery of polyphenols and semi-polar bioactives compared with water alone [79,80]. The observed IC_50_ values (~70–170 µg/mL) fall within ranges reported for fermented tea matrices rather than purified antioxidants. Cardoso et al. (2020) reported substantial variability in antioxidant capacity depending on tea type and fermentation duration [65], while Jakubczyk et al. (2020) similarly noted that kombucha antioxidant activity depends strongly on substrate and fermentation kinetics [64]. Cosmetic-oriented kombucha extracts have demonstrated DPPH IC_50_ values in the 10^2^ µg/mL range [67], supporting that the current magnitudes are realistic for complex fermented systems. Importantly, although antioxidant activity was statistically significant compared with controls, the potency was 4–9 times lower than Trolox or vitamin C. This reinforces that SCOBY extracts represent screening-level antioxidant matrices, not pharmacological equivalents of purified compounds. Such moderate activity is expected given the heterogeneous composition of cellulose-bound fermentation metabolites and residual polyphenols entrapped within the matrix.

The hydroalcoholic extract also exhibited stronger inhibition of α-glucosidase and DPP4 compared with the aqueous extract, again consistent with solvent-dependent extraction of bioactive compounds. Phenolic compounds, flavonoids, and organic acids have been implicated in α-glucosidase inhibition mechanisms [41,42]. Kombucha-based systems have shown similar inhibitory effects in vitro [74], although variability across substrates is substantial. Nevertheless, compared with acarbose and sitagliptin, the SCOBY extracts exhibited markedly higher IC_50_ values (approximately 3–37-fold weaker). This disparity is expected because pharmaceutical inhibitors are optimized for high-affinity binding [68,69]. Reviews consistently emphasize that kombucha-related bioactivities are predominantly supported by preclinical enzyme assays, with limited human evidence [71,73]. Similarly, tyrosinase and elastase inhibition demonstrated measurable but comparatively weak potency relative to kojic acid and ursolic acid. Natural extracts frequently exhibit IC_50_ values in the 10^2^ µg/mL range in these assays [45,75], indicating that the SCOBY-derived activities are directionally consistent with plant-based or fermentation-derived matrices. Collectively, these findings suggest that SCOBY biomass retains biologically active metabolites within its cellulose network. However, the activities should be interpreted as indicative of functional potential rather than therapeutic efficacy.

Across all in vitro assays, the hydroalcoholic extract showed lower IC_50_ values than the aqueous extract; however, this difference should be interpreted cautiously because solvent polarity and extraction temperature were not independently controlled variables. While hydroalcoholic systems are generally more efficient at extracting semi-polar bioactive compounds, the aqueous extraction was performed at 80 °C, which may have induced partial degradation or structural transformation of certain heat-sensitive metabolites. Therefore, the observed differences likely reflect a combined effect of solvent selectivity and thermal processing rather than extraction efficiency alone.

A conceptual linkage can be proposed between the dominant microbial taxa and the observed bioactivities. *Komagataeibacter* spp. drive cellulose biosynthesis and oxidative metabolism, potentially influencing the retention and transformation of polyphenols within the pellicle matrix [35]. Yeasts such as *B. bruxellensis* contribute to ethanol and volatile metabolite production, which may indirectly shape antioxidant profiles through fermentation-mediated biotransformation. However, direct causal inference cannot be made from compositional and enzyme assay data alone. Studies integrating metabolomics with microbial profiling have demonstrated that functional outputs often depend more on metabolic networks than on taxonomic dominance per se [24,26]. Therefore, while the structured community observed here aligns with plausible functional roles, future investigations should incorporate metabolite fingerprinting and quantitative microbial load measurements to establish structure–function relationships.

This study analyzed a single commercial SCOBY sample, which limits generalizability. Kombucha microbial communities vary by substrate, geography, and fermentation practice. Additionally, enzyme-based in vitro assays represent reductionist systems that do not capture bioavailability, digestion, or systemic metabolism. The absence of metabolomic profiling further restricts mechanistic interpretation. It is also important to note that kombucha fermentation is a dynamic process characterized by microbial succession and metabolite evolution over time. The present study analyzed a single time point corresponding to a mature SCOBY pellicle (day 14), and no early-stage comparisons were performed. Therefore, the results reflect the structure and screening-level bioactivities of a stabilized biofilm rather than temporal fermentation dynamics. Despite these limitations, the study provides integrated insight into the species-resolved microbial architecture and screening-level functional properties of a cellulose-rich SCOBY matrix, contributing to the understanding of fermentation-derived biomaterials.

## 4. Conclusions

This study provides an integrated characterization of a kombucha SCOBY pellicle by combining species-level microbial profiling using full-length 16S rRNA and ITS sequencing with a comprehensive panel of in vitro biological assays. The microbial community was highly structured and dominated by AAB, particularly *Komagataeibacter* species, alongside a yeast community overwhelmingly driven by *Brettanomyces bruxellensis*. This configuration is consistent with established SCOBY ecology, in which ethanol-producing yeasts and cellulose-forming AAB operate in a tightly coupled metabolic network.

Functional screening revealed that SCOBY-derived extracts possess measurable antioxidant, antidiabetic-related, and anti-aging-related enzyme inhibitory activities, with hydroalcoholic extracts consistently outperforming aqueous extracts. Nevertheless, the observed IC_50_ values were substantially higher than those of purified reference compounds, emphasizing that the detected activities should be interpreted as preliminary, screening-level bioactivities rather than evidence of pharmacological efficacy.

Collectively, these findings highlight the SCOBY pellicle not merely as a fermentation by-product but as a biologically active biomaterial shaped by a well-defined microbial consortium. The integration of high-resolution microbial data with functional assays contributes to a more holistic understanding of SCOBY structure–function relationships and supports its potential valorization in food, cosmetic, or biomaterial applications. Future studies should focus on detailed chemical profiling, microbial–metabolite linkage analyses, and in vivo or clinical investigations to substantiate functional claims and ensure safety and reproducibility.

## Figures and Tables

**Table 1:** The bacterial community is dominated by *Komagataeibacter saccharivorans* (68,972 reads; 46.28%) and *Acetobacter tropicalis* (22,951; 15.40%), followed by lower-abundance taxa including *Novacetimonas hansenii* (5.17%), *K. rhaeticus* (4.87%), and others each contributing ~1–4%.

Taxa	Read Counts	Relative Abundance (%)
*Komagataeibacter saccharivorans*	68,972	46.28
*Acetobacter tropicalis*	22,951	15.40
*Novacetimonas hansenii*	7710	5.17
*Komagataeibacter rhaeticus*	7261	4.87
*Gluconacetobacter entanii*	5941	3.99
*Komagataeibacter intermedius*	4505	3.02
*Komagataeibacter melomenusus*	3466	2.33
*Acetobacter musti*	3127	2.10
*Pseudomonas luteola*	2104	1.41
*Komagataeibacter swingsii*	1868	1.25

**Table 2:** The top 10 yeast communities are highly dominated by *Brettanomyces bruxellensis* (81.5% of reads), with *Meyerozyma guilliermondii* as the main secondary species (13.31%) and all other taxa each contributing ≤2.47%, indicating strong skew toward a few species.

Species Name	Read Count	Relative Abundance (%)
*Brettanomyces bruxellensis*	107,524	81.5
*Meyerozyma guilliermondii*	17,561	13.31
*Zygosaccharomyces bisporus*	3260	2.47
*Meyerozyma caribbica*	710	0.54
*Brettanomyces anomalus*	468	0.35
*Meyerozyma smithsonii*	286	0.22
*Russula xerophila*	285	0.22
*Pichia kudriavzevii*	204	0.15
*Candida silvatica*	186	0.14
*Pichia ethanolica*	110	0.08

**Table 3:** Antioxidant, antidiabetic, and anti-aging activities of kombucha SCOBY extracts.

Assay	Sample	IC_50_ (µg/mL)	SD (±)	Positive Control	IC_50_ Control (µg/mL)	SD (±)	Significance
DPPH	Hydroalcoholic extract	92.4	4.8	Trolox	21.6	1.2	***
DPPH	Aqueous extract	168.7	7.9	Vitamin C	18.9	0.9	***
ABTS	Hydroalcoholic extract	74.2	3.6	Trolox	15.8	0.7	***
ABTS	Aqueous extract	121.5	6.4	Vitamin C	13.4	0.6	***
α-Glucosidase	Hydroalcoholic extract	112.6	5.3	Acarbose	38.9	2.1	***
α-Glucosidase	Aqueous extract	198.4	9.2	Acarbose	38.9	2.1	***
DPP4	Hydroalcoholic extract	146.8	6.7	Sitagliptin	6.2	0.3	***
DPP4	Aqueous extract	232.1	11.5	Sitagliptin	6.2	0.3	***
Tyrosinase	Hydroalcoholic extract	101.3	4.9	Kojic Acid	12.7	0.6	***
Tyrosinase	Aqueous extract	176.9	8.1	Kojic Acid	12.7	0.6	***
Elastase	Hydroalcoholic extract	128.5	6.1	Ursolic Acid	9.4	0.4	***
Elastase	Aqueous extract	210.6	10.3	Ursolic Acid	9.4	0.4	***

Values are presented as IC_50_ (µg/mL) ± SD. Statistical significance was determined by one-way ANOVA followed by Tukey’s post hoc test comparing extracts with their respective positive controls. Significance levels: *** *p* < 0.001.

## Data Availability

The original contributions presented in this study are included in the article. The datasets presented in this article are not readily available because the data are part of a patent under submission. Further inquiries can be directed to the corresponding author.
